# Blinatumomab in pediatric B-acute lymphoblastic leukemia

**DOI:** 10.3389/fimmu.2025.1611701

**Published:** 2025-07-23

**Authors:** Yifang Cheng, Aiguo Liu

**Affiliations:** ^1^ The Second Clinical School, Tongji Medical College, Huazhong University of Science and Technology, Wuhan, China; ^2^ Department of Pediatrics, Tongji Hospital, Tongji Medical College, Huazhong University of Science and Technology, Wuhan, China

**Keywords:** blinatumomab, pediatric B-ALL, immunotherapy, efficacy, toxicity

## Abstract

Blinatumomab, a bispecific T-cell engager, has demonstrated substantial clinical benefits in treating pediatric patients with relapsed or refractory B-cell acute lymphoblastic leukemia (R/R B-ALL). Approved by FDA for several indications, blinatumomab is now integral to therapeutic protocols for specific pediatric cohorts, with real-world applications steadily increasing. As one of the representatives of cutting-edge immunotherapy for pediatric ALL, blinatumomab plays a crucial role in precision medicine against the backdrop of current genetic testing. Clinical efficacy is influenced by factors such as tumor burden, endogenous T-cell function, CD19 antigen loss, and lineage switch. Treatment-related complications, such as cytokine release syndrome (CRS), neurotoxicity (ICANS), and infections, necessitate vigilant monitoring. Administration involves continuous intravenous infusion, with consideration for drug interactions. Despite proven short-term efficacy and tolerability, long-term impacts on pediatric patients warrant further investigation. Current studies refine dosing strategies and combinational approaches to enhance therapeutic precision for pediatric patients. This review synthesizes selected literature related to clinical trials of blinatumomab, emphasizing determinants of clinical efficacy and adverse events associated with treatment.

## 1 Introduction

Acute lymphoblastic leukemia (ALL) is the most common type of leukemia in children, with precursor B-cell lineage (B-ALL) being the predominant form, accounting for over 75% of all pediatric leukemia (1, 2). As the leading childhood hematologic malignancy, B-ALL is responsible for about one-third of all pediatric cancers. Over the past few decades, the treatment efficacy of pediatric ALL has seen a substantial improvement, largely due to advancements in clinical trials and enhanced supportive care. Survival rates have surged from below 10% in the pre-1970s era to approximately 70% by the 1980s, with current long-term survival exceeding 85% (3–5).

Despite modern therapies achieve cure rates approaching 90%, a subset of pediatric patients continues to encounter challenges such as intrinsic drug resistance or post-remission relapse. For these refractory and relapsed cases, immunotherapy and hematopoietic stem cell transplantation (HSCT) have become main treatment methods. As a representative of immunotherapy, blinatumomab has demonstrated significant efficacy and safety. Current data indicate that the complete remission (CR) rate for refractory or relapsed patients in children treated with conventional chemotherapy is only 20-30%, with a median overall survival (OS) time of only 2 to 4 months (6). In contrast, the CR rate for R/R ALL treated with blinatumomab monotherapy can reach 43-69%, with a median OS time of 6.1 to 13 months (7), which is significantly better than traditional chemotherapy. The efficacy is better with lower tumor burden, and outcomes are even better for MRD+ patients (7).

Blinatumomab functions as a bispecific T-cell engager (BiTE), targeting tumor cells for destruction by simultaneously blinding to tumor-specific antigens (CD19 antigen on malignant B cells) and patients’ own T-cell receptors (typically CD3ϵ) (8). It not only targets tumor cells but also enhances T-cell activity, modifies the tumor microenvironment to reduce immunosuppression, and improves anti-tumor effects. Since its initial application in 2011 on three pediatric patients, blinatumomab has been found to improve disease remission rates and survival rates, effectively clear minimal residual disease (MRD), and offer higher safety compared to cytotoxic drugs through a series of clinical trials. Currently, the focus of pediatric ALL is on precision medicine. Based on the classification of different subtypes of pediatric ALL through genetic testing methods such as Next-Generation Sequencing (NGS), more targeted therapeutic approaches are then adopted, including blinatumomab. Specifically, in several retrospective assessments, children with R/R ALL have shown treatment response rates to blinatumomab ranging from 34-38% to approximately 60% (9). Up to now, almost all published articles indicate that connecting blinatumomab treatment before or after allo-HSCT will improve the survival rate of pediatric patients (9). Furthermore, for children with poor prognosis who have rare genetic variant subtypes, such as germline TP53 mutations and MYC/BCL2 rearrangements, although there is currently limited reported data, blinatumomab represents another potential option beyond cytotoxic drugs (10). In a report regarding nine pediatric patients with TCF3-HLF positive ALL (11), most children experienced durable remissions after using blinatumomab early in the first consolidation as a bridge to HSCT. This rare subtype of childhood ALL is typically characterized by a high rate of treatment failure.

Notably, since its approval by the US Food and Drug Administration (FDA) for the treatment of pediatric ALL, blinatumomab progressively extended its clinical utility. Its favorable efficacy and relative manageable toxicity profile have reshaped treatment paradigms, offering new hope for pediatric patients with R/R ALL (12).

## 2 Clinical adaptations

### 2.1 Official approvals

Blinatumomab (blincyto^®^) has achieved sequential regulatory milestones since its first accelerated approval in 2014 by the US Food and Drug Administration (FDA) for adult and pediatric (≧1month old) patients with relapsed or refractory CD19+ B-cell precursor acute lymphoblastic leukemia (ALL). Subsequent expansion of its indications include:

2018 Authorization: Approval extended to adult and pediatric (≧1month old) patients with CD19+ B-ALL in first or second complete remission exhibiting minimal residual disease (MRD) greater than or equal to 0.1%.
2024 Update: FDA clearance for incorporation into consolidation therapy protocols targeting adult and pediatric (≧1month old) patients with Philadelphia chromosome-negative CD19+ B-ALL during multiphase chemotherapy.

### 2.2 Clinical recommendations

Based on the research and real-world data of blinatumomab in pediatric patients, guidelines from different countries have made relevant recommendations for the application of blinatumomab in pediatric B-ALL.

The 2025, 2nd edition NCCN Guidelines (13) suggest that for newly diagnosed Ph-negative children who achieve a complete response (CR) with minimal residual disease (MRD) positivity after induction therapy, blinatumomab treatment can be recommended, followed by a bridge to allogeneic Hematopoietic Stem Cell Transplantation (allo-HSCT). For high-risk Ph-positive children who fail to achieve CR with induction therapy or still have MRD at the end of consolidation therapy, blinatumomab treatment is also recommended, followed by a bridge to allo-HSCT. For infants with newly diagnosed leukemia accompanied by *KMT2A* rearrangements, the Interfant chemotherapy regime can be recommended, either alone or in combination with blinatumomab, followed by continuation of the Interfant intensive chemotherapy consolidation protocol. For those without *KMT2A* rearrangements, blinatumomab treatment is recommended after induction if MRD is positive, followed by a bridge to allo-HSCT. For children with B-ALL experiencing a first relapse, blinatumomab treatment can be used after achieving CR with induction therapy, regardless of MRD status, with consideration given to a bridge to allo-HSCT. For those who relapse after transplantation, as well as those with multiple relapse or refractory disease, blinatumomab can be used for re-induction therapy.

In the 2024 Chinese Expert Consensus (14), the expert panel’s treatment recommendations are as follows: For newly diagnosed high-risk, chemotherapy-intolerant, and infant patients with leukemia, the use of blinatumomab in combination with chemotherapy for induction of remission and consolidation therapy is recommended; for patients with relapsed/refractory (R/R), the earlier blinatumomab is used, the greater the benefit. Blinatumomab is recommended for salvage therapy of the first relapse and for consolidation therapy in patients with early relapse and positive MRD after induction, corresponding to patients considered as intermediate to high risk. Following this, a bridge to allo-HSCT can lead to longer survival.

### 2.3 Real-world supplementary applications

Since the approval by FDA, blinatumomab has gained widespread recognition for its efficacy in several clinical scenarios. Based on instructions and expert consensus, blinatumomab plays an important role in the real-word clinical treatments and also holds clinical significance in other supplementary situations.

#### 2.3.1 First-line treatment for children

Blinatumomab has been explored as a first-line treatment, particularly for infants with *KMT2A* rearrangements (15). The Interfant-06 study demonstrated a significant improvement in 2-year disease-free and OS rates compared to historical controls (15). Additionally, ongoing clinical trials, such as the AIEOP-BFM ALL 2017 (NCT03643276) and the St. Jude protocols (NCT031177510), as well as the recently finished COG AALL1731 (US), are evaluating its efficacy in the high-risk pediatric B-ALL population. In China, collaborative studies have further extended its application to intermediate-risk cases, marking a shift from its original use in relapse/refractory disease to frontline settings. However, critical questions regarding optimal dosing schedules, treatment duration, and synergistic chemotherapy combinations remain under investigation.

#### 2.3.2 Post-HSCT relapse prevention

Blinatumomab has emerged as a valuable adjunct for preventing relapse following HSCT. When combined with donor lymphocyte infusions, it can effectively help children who are MRD-positive after HSCT to become MRD-negative again. However, its efficacy appears limited in preventing relapses in the central nervous system (16).

#### 2.3.3 Bridging therapy for alleviating chemotoxicity

Blinatumomab has been applied in pediatric ALL patients with severe chemotherapy-related toxicities or those who are intolerant to chemotherapy, and this preliminary exploration has shown a promising outlook. A small proportion of pediatric ALL patients experience overwhelming chemotherapy-related toxicities or temporary contraindications to chemotherapy after receiving chemo, leading to interruptions and delays in chemotherapy or prompting changes in chemotherapy dosages, thereby resulting in treatment failure or relapse. Elitzur et al. (17) reported 11 pediatric patients who received blinatumomab treatment due to severe chemotoxicities, and all patients successfully recovered and transitioned to further therapy. Daniel et al. (18) introduced 15 pediatric ALL cases with invasive fungal disease (IFD) caused by chemotherapy, and these patients received blinatumomab as a bridge treatment, allowing for continued targeted treatment for ALL while recovering from IFD. Another study involving a 10-month-old female patient and a 4-year-old female patient (19) also demonstrated that blinatumomab can improve the toxic state to continue chemotherapy and that blinatumomab treatment is safe even in the presence of infectious complications. In a retrospective analysis conducted by Beijing Children’s Hospital of 23 children treated with blinatumomab (18), 20 were intolerant to chemotherapy, mainly due to pancreatitis, mucositis, cerebral venous thrombosis, infectious shock, and so on. After 1 to 2 cycles of blinatumomab treatment, all children achieved molecular biological remission with negative MRD. Among them, 4 children with relapse subsequently underwent HSCT, and the remaining children received maintenance therapy. Blinatumomab bridge therapy shortens the duration of chemotherapy interruption and provides a novel treatment option for pediatric ALL patients who cannot tolerate cytotoxic therapy. However, experience and data on the use of blinatumomab in pediatric patients with severe chemotherapy-related toxicities are limited, and prospective clinical studies are needed to determine the exact and optimal role of blinatumomab in improving treatment and reducing treatment-related toxicities.

## 3 Treatment response across different subtypes

With the continuous advancement of molecular technology and the application of NGS technology, both the International Consensus Classification (ICC) and the World Health Organization (WHO) have conducted detailed molecular subtyping of B-ALL. Although this has increased the complexity of subtyping diagnosis, it has significant implications for personalized precision treatment and prognostic management. Comprehensive genomic analysis of large cohorts of ALL, through the identification of novel clonal, subtype-defining chromosomal alterations, has reduced the proportion of patients previously classified as “others” from 25% to approximately 5% (20), thereby expanding the scope of precision medicine treatment for pediatric ALL. Children with different subtypes of B-ALL harbor distinct abnormal molecular signaling pathways or other biological pathways, which correspond to varying degrees of prognosis. The identification of clear subtypes provides definite abnormal targets, facilitating the selection of targeted drugs and offering the opportunity for preemptive treatment planning for subtypes with poor prognosis. There is currently evidence that ETV6-RUNX1, high hyperdiploidy of chromosomes 4, 10, and 17, or double/triple trisomies are associated with a favorable prognosis, whereas hypodiploidy, BCR-ABL1, KMT2A rearrangements (KMT2AR), TCF3-HLF, and intrachromosomal amplification of chromosome 21 are associated with an adverse prognosis (21). Recently identified novel subtype-defining chromosomal alterations, some of which have prognostic and/or therapeutic implications, may involve multiple rearrangements of a single partner gene, sequence mutations of transcription factors, or a spectrum of genomic alterations within a single group (20), such as the MYC rearrangement subtype, which has an extremely poor prognosis.

Meanwhile, the molecular subtypes also provide a platform for understanding the genetic basis and clonal architecture of R/R B-ALL, contributing to the progress in the mechanisms of relapse. The mutations in relapsed ALL often originate from minor clones that exist at diagnosis, which survive therapy and acquire additional cooperating mutations, thereby becoming the founding clones of relapse (22). These founding clones may arise as a result of chemotherapy-induced selection, and thus are drug-resistant, rendering the original chemotherapy ineffective. Targeted therapies, such as tyrosine kinase inhibitors (TKIs), can effectively inhibit tumor progression and exert antitumor effects by interfering with molecular signaling pathways, but they face the problems of drug resistance and relapse. At this point, immunotherapy, which is not dependent on specific genetic abnormalities, can overcome the chemotherapy-resistant mutations that are enriched in relapsed ALL (22). Specifically, blinatumomab bridging to HSCT has demonstrated high efficacy and low toxicity in children with intermediate and high-risk first relapse of B-ALL (23).

### 3.1 B-ALL with BCR::ABL1 fusion

The BCR::ABL1 fusion gene is generated by a reciprocal translocation between chromosomes 9 and 22, which results in the formation of the Philadelphia chromosome (Ph). Pediatric Ph+ B-ALL is a subtype with a poor prognosis, accounting for 2%-5% of childhood ALL (24). The BCR-ABL1 fusion event leads to the abnormal activation of tyrosine kinase, which in turn causes the dysregulation of its downstream pathways. Therefore, the application of TKIs has brought significant improvement for children, with the survival rate of pediatric Ph+ ALL achieving a leap from 20% to over 60% (25). At present, the treatment of pediatric Ph+ ALL has formed a comprehensive strategy centered on the combination of TKIs and chemotherapy, and is gradually evolving towards precision stratification and targeted immunotherapy. Blinatumomab has been applied in the consolidation phase of children with Ph+ ALL and in those with relapsed/refractory disease. In patients with relapsed/refractory Ph+ B-ALL, blinatumomab monotherapy has demonstrated a high CR rate and molecular complete remission (CMR) rate. Besides, the RIALTO study showed that blinatumomab had a significant effect on MRD remission, with an MRD remission rate of 79% in children with a baseline blast count of ≥5%, and an MRD remission rate as high as 92% in children with a baseline blast count of <5%.

### 3.2 B-ALL with BCR::ABL1-like features

BCR::ABL1-like ALL, also termed as Ph-like ALL, is a high-risk B-ALL, which is characterized by adverse clinical features and a poor relapse-free survival rate, even when treated with risk-adapted multi-agent chemotherapy regimens. The advent of NGS technology has unveiled the diversity of kinase-activating genetic drivers in Ph-like ALL, which may be amenable to “personalized” molecularly targeted therapies. Ph-like ALL is characterized by a variety of kinase-activating alterations, leading to a gene expression profile similar to that of Ph+ ALL, but lacking the typical BCR::ABL1 fusion. The proportion of this subtype in pediatric B-ALL is 10-13%. Blinatumomab is primarily used in the treatment of pediatric Ph-like ALL for continuous administration during the consolidation phase until minimal residual disease (MRD) is negative, followed by allogeneic hematopoietic stem cell transplantation (allo-HSCT), which helps to improve the remission rate and survival rate.

### 3.3 Novel molecular subtypes

The molecular heterogeneity of B-ALL is far more complex than previously recognized. As research continues to delve deeper, an increasing number of novel molecular subtypes are emerging. The discovery of these new subtypes further expands the boundaries of our understanding of the molecular characteristics of B-ALL and also brings new opportunities and challenges for future therapeutic strategies. This review mainly focuses on the rare (with a frequency of only 1- 9%) but extremely poor-prognosis MYC-rearranged subtype. This subtype was initially described in Burkitt lymphoma (BL). In ALL, it represents a rare molecular subtype characterized by MYC rearrangement, positive expression of TdT, optional CD34 expression, frequent absence of surface immunoglobulin (sIg) and CD20, and potential Burkitt-like morphological features (26). In children with this subtype of B-ALL, the MYC gene is typically overexpressed. Studies have shown that intrinsic defects in the B-ALL microenvironment lead to reduced production of type I interferons (IFN-Is) by plasmacytoid dendritic cells and/or autocrine IFN-Is from B cells, resulting in impaired IFN-I-driven immune responses that promote tumor progression in the MYC subtype (27). The abnormality of IFN-Is further diminishes IL-15 transcription, leading to impaired maturation of natural killer (NK) cells in the microenvironment. Consequently, these NK cells cannot lyse NK cell-sensitive targets as efficiently as normal NK cells. An increased frequency of abnormal NK cells is independently associated with heightened disease severity and poor prognosis in patients (28). Meanwhile, MYC overexpression enhances the sensitivity of B-ALL cells to NK cell-mediated cytotoxicity. Thus, NK cells secreting IL-15 may serve as a therapeutic approach for the MYC subtype, a hypothesis validated in *in vitro* experiments (27). Regarding blinatumomab, while current research has not proven its specific efficacy against this subtype, its mechanism of action—promoting the release of various cytokines to modulate the immune microenvironment—suggests that if used during the consolidation phase, it could effectively maintain the activity and quantity of IL-15-secreting NK cells. Blinatumomab has synergistic effects with NK cell therapy, which may enhance treatment tolerance in children and reduce therapy-related toxicity. Ocadlikovad et al. found that after treatment with blinatumomab, there was a persistent increase in NK cells, such as the cytotoxic CD56dim NK cell subset, but this upregulation was only observed in peripheral blood, not in the bone marrow (29). The mechanism behind this upregulation of NK cells is not clear and may be related to the off-target effects of blinatumomab (29). Blinatumomab, as one of the cutting-edge immunotherapeutic modalities, can be combined with targeted therapy in rare subtypes such as MYC-rearranged to enhance efficacy and treatment safety. However, due to the limited number of clinical samples, further studies are needed to confirm this. International cooperation to design prospective clinical trials can be carried out to achieve this goal.

## 4 Factors impact on efficacy

Clinical evidence demonstrates superior efficacy of blinatumomab compared to conventional chemotherapy in relapsed/refractory B-ALL. A phase III multicenter randomized clinical trial reported significantly improved outcomes with blinatumomab, including 2-year overall survival (OS) rates of 81% versus 56% with chemotherapy, and MRD remission rates of 93% versus 24% after one treatment cycle (30). Extended follow-up data revealed that patients receiving blinatumomab consolidation therapy maintained event-free survival (EFS) exceeding 50% and OS surpassing 80% at 57 months, with consistent hazard ratios 0.33 for both EFS (95%CI: 0.19 - 0.59) and OS (95% CI: 0.15 - 0.72) compared to chemotherapy controls (30). Blinatumomab provides higher health benefits in treating R/R ALL compared to traditional chemotherapy. Blinatumomab has a clear clinical significance, with prominent therapeutic effects, filling a gap in clinical treatment, achieving rapid and high-quality hematological remission, effectively clearing MRD, offering more HSCT possibilities for patients, and improving their long-term survival. Its efficacy has been verified in adult patients in China.

Blinatumomab treatment responses exhibit interpatient variability influenced by complex multifactorial interactions. However, because of the complex interplay between external environmental factors and leukemia-intrinsic factors, the predication of efficacy remains limited. Unlike conventional chemotherapy regimens where treatment efficacy correlates with established predictors including patient’s age, duration of prior remission, chemosensitivity profiles, and post-transplant relapse status, these conventional parameters demonstrate limited predictive value for blinatumomab outcomes (31). The observation that traditional efficacy prediction indicators do not match the response to blinatumomab is consistent with the fact that blinatumomab works by CD3/CD19 bispecific targeting to lyse tumor cells, thereby bypassing many mechanisms associated with chemotherapy resistance (32). Therefore, traditional indicators for predicting chemotherapy efficacy are not applicable for predicting the efficacy of blinatumomab. Currently, new biomarkers are being explored to better predict the efficacy of blinatumomab. Apart from the known T-cell subsets and CD19 status, some new molecular markers (such as specific gene mutation or immune cell surface markers) may help identify patients who are more likely to benefit from the treatment. Tumor burden, the function of endogenous T-cell and status of T-cell subset, loss/decrease of CD19 antigen are important factors affecting the efficacy of blinatumomab. The rare phenomenon of lineage switch can also lead to treatment failure. Moreover, the efficacy may also be influenced by the drug’s specific impact on particular patients and individual differences among patients, including genetic background, immune status, and prior treatment history.

### 4.1 Tumor burden

Tumor burden serves as an important clinical indicator for assessing disease severity and predicting therapeutic outcomes in pediatric B-ALL. This quantitative measure reflects both the absolute number and anatomical distribution of malignant cells within the patient’s hematopoietic system. In clinical practice, tumor burden assessment employs a multimodal diagnostic approach incorporating the MICM model (morphological examination, immunophenotypic characterization, cytogenetic analysis, and molecular genetic profiling), complemented by bone marrow aspiration and biopsy procedures, comprehensive immunophenotyping panels, advanced genetic testing methodologies, and sensitive minimal residual disease (MRD) monitoring techniques. These diagnostic tools collectively provide a robust framework for accurate disease quantification and characterization. Current risk stratification protocols universally incorporate tumor burden measurements as a key determinant of disease classification. The National Cancer Institute (NCI) risk stratification system categorizes patients into two distinct groups: standard-risk and high-risk, based on predefined tumor burden thresholds. Alternative classification systems employed by various international cooperative groups further refine this approach by implementing three-tiered stratification schemes (low-risk, intermediate-risk, and high-risk categories). These risk-adapted classifications serve critical functions in clinical management by guiding therapeutic intensity selection, informing prognostic predictions, and facilitating comparative outcome analyses across treatment protocols and clinical trials.

Extensive clinical investigation has established a strong inverse correlation between baseline tumor burden and treatment efficacy. Several prospective studies and retrospective analyses have consistently demonstrated that pediatric patients presenting with lower initial disease burdens achieve significantly higher rates of complete hematological remission following blinatumomab therapy (30, 32, 33). This relationship extends to long-term clinical endpoints in adult patients, with lower tumor burden cohorts exhibiting superior relapse-free survival and overall survival rates compared to their high-burden counterparts (34). The biological underpinnings of this clinical observation involve several interrelated mechanisms. From a pharmacological perspective, the bispecific T-cell engager mechanism of blinatumomab requires adequate T-cell to tumor cell ratios for optimal cytotoxic activity. Excessive leukemic cell populations may overwhelm endogenous T-cell effector capacity through numerical superiority and potential immune exhaustion phenomena, thereby limiting therapeutic effectiveness. Meanwhile, the rapid cytoreduction characteristic of blinatumomab therapy in high tumor burden patients precipitates substantial cellular destruction, triggering massive release of intracellular contents and proinflammatory cytokines. This pathophysiological cascade manifests clinically as an increased incidence and severity of cytokine release syndrome (CRS), a potentially life-threatening treatment complication (35). Furthermore, the abrupt liberation of cellular metabolites from lysed leukemic cells may overwhelm normal homeostatic mechanisms, resulting in tumor lysis syndrome (TLS) characterized by dangerous electrolyte disturbances and acute kidney injury (36). Adverse reactions have a negative impact on the treatment effect and reduce the safety of treatment. In response to these challenges, contemporary treatment algorithms have incorporated strategic pretreatment approaches for high tumor burden patients in order to make the treatment process safer, enable patients to better tolerate the treatment, and improve treatment compliance and the overall therapeutic effect. Clinical evidence from adult populations demonstrates that preliminary cytoreduction with conventional chemotherapy or targeted debulking regimens prior to blinatumomab initiation significantly reduces the incidence and severity of CRS events while simultaneously improving rates of MRD negativity (37). In pediatric patients, individuals with a higher tumor burden may require more aggressive pretreatment to improve the treatment efficacy. The development of refined tumor burden assessment techniques and corresponding treatment algorithms continues to represent an active area of clinical investigation in pediatric B-ALL management.

### 4.2 Endogenous T-cell function and T-cell subset impact

Blinatumomab activates T cells by targeting them, thereby attracting leukemic cells. After the use of this frug, the patient’s immune system is activated, capable of activating different T-cell subset. Although blinatumomab can activate T cells, the patients’ immune status, such as the basal function and number of T cells, will still affect the treatment outcome. Growing evidence suggests that endogenous T-cell function and T-cell subsets influence the response to blinatumomab immunotherapy. The baseline functionality of endogenous T cells, such as their ability to produce cytokines like IFN-γ upon initial exposure to antigens, can significantly impact how well blinatumomab works. T cells with higher pre-treatment IFN-γ production were associated with a more robust anti-leukemia response after blinatumomab treatment (38).

Recent single-cell transcriptomic studies have provided comprehensive insights into the complex immunological mechanisms underlying blinatumomab’s therapeutic effects in B-ALL. These investigations have identified four different T-cell subsets activated by blinatumomab, including CD8+ effector memory T cells (TEM), CD4+central memory T cells (TCM), naïve T cells, and regulatory T cells (Tregs). Detailed analysis of gene expression patterns in these activated clusters have revealed significant upregulation of multiple critical pathways, including immune system activation, glycolytic metabolism, interferon-alpha (IFNA) signaling, gap junction communication, and interferon-gamma (IFNG) signaling pathways, reflecting the multifaceted nature of T-cell activation induced by blinatumomab therapy (39). The activation of these T-cell populations following blinatumomab administration leads to substantial production of proinflammatory cytokines, which mediates the drug’s therapeutic effects. Among these subsets, CD8+ TEM cells demonstrate particularly robust activation, exhibiting markedly higher expression of cytotoxic factors such as perforin (PRF1), interferon-gamma (IFNG), and FAS ligand (FASLG), along with numerous cytokines and chemokines such as CCL2, CCL3, CCL3L1, CCL4 and TNFSF9 compared to other T-cell subsets (39). This distinct cytokine secretion profile suggests that different T-cell populations contribute variably to target cell lysis, thereby influencing the overall treatment efficacy in a subset-specific manner. CD4+ TCM cells, for example, are crucial for maintaining a long-term immune response. They can rapidly proliferate and differentiate into effector cells upon re-encountering the antigen. Patients with a higher proportion of CD4+ TCM cells at the start of treatment are more likely to achieve long-term remission, suggesting their importance in sustaining the anti-leukemia immune attack (38). Interestingly, transcriptomic analysis of responding patients has revealed enrichment of tumor cell immune response genes, suggesting that the efficacy of blinatumomab-induced T-cell activation may be modulated by leukemia-intrinsic factors (31). Furthermore, clinical observations have identified that increased frequencies of Tregs in peripheral blood can predict *in vitro* response to blinatumomab, likely mediated through interleukin-10-dependent suppression of T-cell activity (31). Additional investigations focusing on Tregs, specifically those identified by CD4, CD25 and FOXP3 expression makers, have demonstrated that blinatumomab-activated Tregs promote immunosuppressive effects through IL-10 production, which subsequently inhibits general T-cell proliferation and reduces CD8+ T-cell-mediated lysis of ALL cells, ultimately impacting treatment outcomes (40). Understanding these complex interactions between different T-cell subsets and blinatumomab can potentially lead to more personalized treatment strategies for patients.

### 4.3 CD19 antigen loss/decrease

The CD19 antigen is the target site for blinatumomab’s action. However, leukemic cells may develop resistance through either complete loss of CD19 expression or significant reduction in antigen density, thereby impairing T-cell recognition and cytotoxic attack against malignant cells. This immune evasion mechanism can manifest as either complete immunological escape or progressive T-cell exhaustion, both of which substantially compromise treatment outcomes. A comprehensive retrospective analysis of real-world data from adult patients receiving blinatumomab revealed that 9.8% of cases experienced relapsed with CD19-negative disease, representing 34.2% of all relapsed events, with similar patterns observed in pediatric patients (41). These findings confirm CD19 antigen loss as a major pathway for leukemic cells to evade CD19-directed immunotherapies. Analysis of patient samples with antigen loss after blinatumomab treatment conclude that possible mechanisms leading to CD19 antigen loss include acquired mutations in the CD19 gene itself, alterations in CD81 (a crucial chaperone protein required for CD19 membrane expression), and other chromosomal causes (31). In addition, a high tumor burden is independently associated with CD19 loss and is related to a poor EFS (42). This relationship suggests that patients presenting with extensive disease may be at heightened risk for developing this resistance mechanism during treatment. The clinical impact of CD19 loss has been extensively documented across pediatric studies. A comprehensive single-center retrospective analysis incorporating data from multiple trials confirmed that diminished or absent CD19 expression represents a major contributor to treatment failure in pediatric B-ALL (30). Across various pediatric cohorts, a substantial proportion of poor responders exhibited either reduced CD19 antigen density or complete antigen loss, with this phenomenon being strongly associated not only with diminished initial response rates but also with increased risk of disease recurrence. These observations underscore the universal significance of CD19 antigen modulation as a key determinant of treatment outcomes across all age groups. Further complicating this picture, pediatric patients demonstrating poor response to blinatumomab frequently exhibit concurrent upregulation of T-cell exhaustion markers, particularly PD-1 and TIM-3 (43). This dual phenomenon of CD19 loss combined with T-cell exhaustion creates a synergistic immunosuppressive environment that further facilitates leukemic cell escape from immune surveillance. The co-occurrence of these mechanisms suggests a potential feedback loop where CD19 loss reduces antigenic stimulation while exhaustion markers dampen remaining T-cell activity, collectively crippling the anti-leukemic immune response. However, t the clinical consequences of CD19 loss appear somewhat less severe in blinatumomab therapy compared to CD19-directed CAR-T cell treatments (44). This differential impact stems from fundamental mechanistic distinctions between these immunotherapeutic approaches. CAR-T cells rely exclusively on direct CD19 recognition for target cell engagement, making them particularly vulnerable to antigen loss variants. In contrast, bispecific antibody design of blinatumomab may retain partial efficacy even in the face of CD19 modulation, as its T-cell activating capacity persists independently of absolute antigen density. This relative advantage may explain why some patients with partial CD19 loss can still derive clinical benefit from blinatumomab despite suboptimal responses.

### 4.4 Lineage switch

Lineage switch is a rare phenomenon observed in patients after receiving blinatumomab treatment, where lymphoid tumor cells transdifferentiate into myeloid tumor cells that do not express CD19. The phenomenon is particularly well-documented in cases harboring mixed lineage leukemia (MLL) rearrangements, where the leukemic cells demonstrate inherent lineage plasticity and may undergo myeloid conversion under therapeutic pressure. While lineage switching has been historically associated with conventional chemotherapy regimens, emerging evidence confirms its occurrence following blinatumomab treatment, with the most common transformation being to acute myeloid leukemia (AML) (45). A comprehensive multicenter study involving 182 pediatric BCP-ALL patients treated with blinatumomab provided detailed insights into the incidence and molecular characteristics of this phenomenon (46). The investigation identified six confirmed cases of lineage switch occurring either during active blinatumomab treatment or in the post-therapy period. These cases represented 17.2% (4/23) of all documented treatment-resistant instances and 3.2% (2/63) of relapse events, establishing lineage conversion as an important mechanism of therapeutic failure. The phenotypic manifestations of lineage switch exhibited considerable heterogeneity among affected patients. Approximately half of the cases demonstrated complete conversion from BCP-ALL to CD19-negative AML, while the remainder displayed more complex immunophenotypic patterns characterized by the coexistence of residual CD19-positive B lymphoblasts with newly emergent CD19-negative blast populations of either myeloid or unclassifiable lineage. The transdifferentiated myeloid tumor cells no longer express CD19, thereby evading the targeted therapy of blinatumomab and leading to a decrease in treatment efficacy. The mechanisms of lineage switch are currently unclear and may be related to cytogenetic abnormalities (45).

### 4.5 Other factors

Several additional clinical considerations also impact the therapeutic effectiveness of blinatumomab in B-ALL management. The drug’s pharmacokinetic properties, particularly its central nervous system (CNS) penetration capabilities and activity against extramedullary disease, represent important determinants of clinical outcomes. In the ALL1331 clinical trial, it was indicated that the efficacy of blinatumomab within the CNS may be limited, leading to poorer prognosis for patients with isolated CNS disease. Additionally, the efficacy of blinatumomab in extramedullary sites may be limited, resulting in a poorer prognosis for low-risk patients with isolated extramedullary relapse, especially those with isolated CNS disease. In actual treatment, Patient tolerance represents another critical factor influencing blinatumomab treatment success. Clinical experience has shown that adverse event profiles frequently necessitate dose modifications or temporary treatment interruptions, potentially compromising therapeutic efficacy. Optimizing the dose adjustment strategy and managing adverse reactions, patients’ tolerance and treatment compliance can be improved, thus enhancing the overall efficacy. Pediatric and adult patients have different tolerances to the drug. Pediatric patients generally have better tolerance to immunotherapy but this enhanced tolerance coexists with unique vulnerabilities, including increased susceptibility to specific developmental toxicities such as growth impairment and delayed maturation processes (9). Moreover, the strategic positioning of blinatumomab within comprehensive treatment algorithms represents an additional variable affecting clinical outcomes. Emerging evidence supports multiple effective sequencing approaches for blinatumomab administration in R/R ALL management. The agent has demonstrated significant utility when employed as consolidation therapy following successful induction remission, where it may deepen molecular responses and prolong remission duration. Alternatively, pre-transplant administration has shown efficacy in reducing tumor burden prior to allo-HSCT, thereby potentially enhancing engraftment success rates and reducing post-transplant relapse risk (37). Blinatumomab has also been successfully incorporated as a bridging therapy preceding CAR-T cell interventions, where its tumor-reducing effects can create more favorable conditions for subsequent cellular therapy, improving both safety profiles and treatment success rates (37).

## 5 Toxicity

Current clinical trials indicate that the toxicity of blinatumomab, the adverse events (AE) produced in clinical applications, is less than that of traditional chemotherapy in general. The most common adverse reactions include fever, headache, infection, and febrile neutropenia, with fever being the most common AE at the recommended dose (80%) (47). Other more common side effects include dizziness, tremors or ataxia, nausea, hypokalemia, fatigue, constipation, and diarrhea. More serious AE include cytokine release syndrome (CRS) and neurological AE, which often require immediate discontinuation of the drug and corresponding treatment. Although blinatumomab is generally well-tolerated, serious adverse reaction such as CRS, immune effector cell-associated neurotoxicity syndrome (ICANS) and infections have been identified in clinical trials and real-world studies, necessitating discontinuation of the drug.

### 5.1 cytokine release syndrome

CRS is considered a clinically significant systemic inflammatory response associated with blinatumomab immunotherapy (48), mediated by elevated levels of cytokines and other inflammatory markers. CRS is characterized by fever and multi-organ dysfunction. The NCCN 2025 second edition describes CRS as a spectrum of clinical symptoms ranging from fever or hypothermia in mild cases to potentially life-threatening hypotension and end-organ damage in severe manifestations (13). The American Society for Transplantation and Cellular Therapy (ASTCT) has established a standardized five-grade classification system for CRS severity, with grade 1 representing mild febrile reactions and grade 5 indicating fatal complications requiring immediate intervention (49). Clinical management strategies vary according to severity, with grades 1–2 typically managed through symptomatic support, while grades 3–5 necessitate treatment interruption combined with corticosteroids, vasopressors, and IL-6 receptor antagonists such as tocilizumab following manufacturer guidelines. Epidemiological data indicate that CRS occurs in 4-22% of pediatric patients receiving blinatumomab, though high-grade (≥3) events are less frequent (approximately 3%) (29). Interestingly, a 2022 meta-analysis found comparable CRS incidence rates between blinatumomab and conventional chemotherapy groups when evaluating pediatric safety profiles (12).

The pathophysiological mechanisms underlying CRS involve complex cytokine networks activated during blinatumomab therapy. When the bispecific antibody engages T-cells with CD19+ leukemic cells, massive T-cell activation triggers an exaggerated release of proinflammatory mediators including IFN-γ, IL-6 and TNF. These cytokines normally help with immune response, but in CRS, their release far exceeds physiological levels, leading to a systemic inflammatory response. Cytokines such as IFN-γ and GM-CSF can further stimulate macrophages and monocytes to release more IL-1 and IL-6. IL-6 plays a key role in CRS. It nor only directly mediates acute inflammatory response but also induces the expression of Vascular Endothelial Growth Factor (VEGF), increasing vascular permeability and leading to capillary leak and hemodynamic instability (50). CRS is a target phenomenon associated with multiple cytokines, most notably IFN-γ and IL-6, with a lesser association with TNF (51). Several risk factors influence CRS development, with baseline tumor burden representing a particularly important modifiable predictor. Clinical evidence confirms that cytoreductive strategies implemented prior to blinatumomab initiation can mitigate both CRS incidence and severity (51). Most CRS is reversible, and effective prevention can be achieved by identifying high-risk patients before blinatumomab administration, premedication with dexamethasone, and stepwise dose escalation. After CRS occurs, most patients can continue blinatumomab treatment after CRS subsides by interrupting blinatumomab therapy, administering corticosteroids and IL-6 receptor antagonists according to graded assessment, and/or supportive care (50). However, accurate diagnosis remains challenging due to significant symptom overlap with other conditions including infusion reactions, systemic infections, capillary leak syndrome, and hemophagocytic lymphohistiocytosis/macrophage activation syndrome (14). The NCCN 2025 guidelines emphasize the importance of thorough infectious disease evaluation in suspected CRS cases, recommending empirical antimicrobial therapy when appropriate given the potential for concurrent severe infections to mimic CRS presentation (13). This diagnostic complexity underscores the need for comprehensive clinical assessment and multidisciplinary management approaches when addressing potential CRS events during blinatumomab treatment.

### 5.2 Immune effector cell-associated neurotoxicity syndrome

Immune effector cell-associated neurotoxicity syndrome (ICANS) represents a clinically significant neurological complication observed in patients undergoing T-cell activating immunotherapies such as blinatumomab for B-cell malignancies. This neuropsychiatric syndrome, first formally characterized by the ASTCT in 2019, typically manifests during the initial treatment cycle with symptom duration varying from transient to prolonged depending on severity (48). The clinical presentation encompasses a spectrum of neurological disturbances including confusion, dysphasia, somnolence, ataxia, tremors, seizures, and syncopal episodes. Neurological adverse events are common in therapies that utilize activated T cells to destroy malignant B-cell tumors, often occurring in the early stages of the first treatment cycle, with short symptom duration and most being reversible. ICANS is a common and potentially life-threatening adverse reaction associated with T-cell involvement in immunotherapy. These symptoms reflect the complex interplay between activated immune effectors and the central nervous system, with pathophysiological mechanisms that may overlap with concurrent cytokine release syndrome (CRS) while maintaining distinct clinical features (52). Blinatumomab, by activating T cells, leads to the release of a large number of cytokines. These cytokines may disrupt the blood-brain barrier, exposing brain tissue to circulating cytokines and thereby inducing neurotoxicity. Cytokines like IL-6 may affect the integrity of the blood-brain barrier, leading to brain tissue edema and neurological dysfunction. Additionally, activated T-cells themselves may transmigrate across the compromised blood-brain barrier, establishing localized inflammatory foci within the CNS parenchyma that further exacerbate neurotoxicity (52). These mechanisms collectively contribute to the diverse neurological manifestations observed in clinical practice. The ASTCT has established a standardized five-tier grading system for ICANS severity assessment (49). Grade 1 events typically involve mild symptoms such as headache or subtle tremors, while grade 5 represents life-threatening complications including status epilepticus or cerebral edema. Clinical management strategies are severity-dependent, with grades 1–2 generally managed through supportive measures and close monitoring, whereas grades 3–5 necessitate immediate treatment interruption combined with high-dose corticosteroids and other neuroprotective interventions. Epidemiological analyses reveal important age-related differences in ICANS presentation and outcomes. Pediatric populations demonstrate lower overall incidence rates (3.7-24%) compared to adults, with severe (grade ≥3) events occurring in only 2-3.6% of cases (29). However, children exhibit distinct clinical characteristics including earlier symptom onset and more rapid progression timelines, potentially increasing acute life-threatening risks despite lower absolute frequencies (33). A comprehensive 2022 systematic review of published clinical trials demonstrated comparable seizure risks between blinatumomab and conventional chemotherapy, but identified significantly higher encephalopathy rates with blinatumomab-based immunotherapy (53). These findings highlight the need for age-specific monitoring protocols and management algorithms. ICANS is one of the common reasons for discontinuing blinatumomab therapy, and the drug should be stopped immediately upon the appearance of grade ≥3 neurological symptoms, followed by appropriate treatment. Due to the short elimination half-life of blinatumomab, most neurotoxic symptoms can disappear after discontinuation of the drug and initiation of steroid therapy. Seizures are a relatively rare symptom, and the use of antiepileptic drugs should be cautious, with routine use of antiepileptic drugs for prophylaxis not recommended. To prevent the occurrence of ICANS, it is first necessary to identify high-risk patients, such as those with a high tumor burden or a history of neurological disease, and to adopt more cautious treatment strategies for these patients. Before treatment, corticosteroids such as dexamethasone can be used for pre-treatment, and a stepwise dose-escalation approach can be employed to reduce the risk of ICANS occurrence.

### 5.3 Infections

Infections is currently one of the most significant adverse reactions in patients receiving blinatumomab treatment. Patients with leukemia have various risk factors for infection, including immunosuppression, hematological toxicity, concomitant use of immunosuppressants, and catheter-related infection. As an immunomodulatory antibody, blinatumomab may suppress the immune functions of B cells and T cells, leading to hypogammaglobulinemia and immune dysregulation, thereby increasing the risk of infection. Blinatumomab has myelosuppressive effects, causing persistent cytopenia, and may also lead to B-cell aplastic anemia; meanwhile, treatment-related neutropenia is a common phenomenon in immunotherapy, making patients more susceptible to infections. However, blinatumomab’s myelosuppressive effect is weaker than traditional chemotherapy, and the suppression is mostly transient. The patient’s weakened immune system, coupled with the use of corticosteroids or tocilizumab for infection. Additionally, since blinatumomab is typically administered through long-term continuous infusion, requiring the establishment of a venous infusion pathway, catheter-related infections must also be vigilantly monitored. To prevent severe infections or life-threatening conditions, routine blood tests should be conducted for pediatric patients, and attention should be paid to the emergence of infection-related clinical symptoms. If symptoms and signs of suspected infection appear, empirical antimicrobial treatment should be initiated immediately, and pathogen testing should be completed as soon as possible.

## 6 Administration

The clinical management of blinatumomab is decisive for its efficacy and the incidence and severity of adverse events. The appropriate route of administration is determines based on pharmacokinetic characteristics and the patient’s specific condition, a course of treatment is selected and planned, and adverse events that occur after medication are managed.

### 6.1 Route of administration and course of treatment selection

In studies conducted over 4~8 hours under continuous intravenous infusion, it was confirmed that blinatumomab exhibits linear pharmacokinetic characteristic, which means that its clearance rate and distribution volume remain constant across different dosage ranges. The average systemic clearance of 2.92 L/hour reflects rapid elimination from circulation, while the average volume of distribution of 4.52 L confirms predominantly intravascular compartmentalization. These predictable pharmacokinetic parameters contribute significantly to both the therapeutic efficacy and safety profile of blinatumomab in clinical applications. The standard administration protocol for both adult and pediatric patients involves continuous intravenous delivery using precision infusion pumps to maintain constant flow rates. Particular attention must be given to pediatric dosing regimens to minimize adverse events while maintaining therapeutic effectiveness. For pediatric patients with body weight below 45 kg, a carefully titrated dose-escalation approach is implemented, typically progressing through 5, 10, and 15 μg/m² dose levels with close monitoring for toxicity. Patients weighing 45 kg or more receive fixed dosing according to established protocols. The conventional treatment cycle consists of 4 weeks of continuous infusion followed by a 2-week treatment-free interval, a schedule designed to achieve and maintain therapeutic serum concentrations while allowing for physiological recovery. Recent clinical investigations have explored alternative administration routes to potentially improve treatment convenience and accessibility. A multicenter phase 1b trial expansion cohort evaluated subcutaneous blinatumomab administration in adults with relapsed/refractory B-ALL, demonstrating both feasibility and acceptable safety profiles with this delivery method (54). The subcutaneous route offers potential advantages in outpatient management and reduced healthcare resource utilization. However, it is important to note that comparable studies in pediatric populations have not yet been conducted, and intravenous infusion remains the only approved administration method for children at present. This represents an important area for future clinical investigation, particularly given the potential benefits of subcutaneous administration in pediatric oncology care settings.

### 6.2 Drug interactions

Blinatumomab had drug interactions with other medications, which may affect its efficacy or lead to adverse drug events. Common drug interactions include the following. Blinatumomab can increase blood glucose levels, so when used with glucose-lowering agents or insulin, it is necessary to carefully monitor blood glucose levels and adjust medication doses as needed. The use of white blood cell growth factors (such as filgrastim) in combination with blinatumomab may increase the risk of severe infection during treatment. Sedatives, hypnotics, or anesthetic drugs used in conjunction with blinatumomab may increase the risk of adverse reactions such as somnolence, fatigue, dizziness, and confusion. As an immunotherapy, blinatumomab may interact with other immunosuppressants such as cyclosporine, tacrolimus, and methotrexate. These immunosuppressants may reduce the immunostimulatory effects of blinatumomab, thereby weakening its therapeutic effect. Therefore, when treating with blinatumomab, it is important to carefully consider other medication choices and conduct monitoring.

## 7 Conclusion

Blinatumomab is the world’s first and only approved BiTE therapy drug. One end is bound to CD19 expressed on the surface of B cells, and the other end to CD3 expressed on the surface of T cells, activating T cells and enabling them to exert cytotoxic effects, thus lysing B lymphoid leukemic cells. It is used for the treatment of adult and pediatric B-ALL. As clinical researches continue to advance, the clinical application of blinatumomab is expanding, and the management of its side effects is becoming increasingly refined. Meanwhile, efforts are being made to further clarify the factors affecting efficacy and to optimize treatment plans or adopt combination therapy strategies, in order to better ensure its therapeutic effectiveness. In the application of treating pediatric B-ALL, blinatumomab has shown significant efficacy and safety compared to traditional chemotherapy in treatments such as R/R B-ALL and MRD clearance. However, due to the limitations of pediatric clinical research duration, the long-term effects of treatment in children are currently unclear. Currently, blinatumomab is being explored as part of first-line treatment regimen, especially in “chemotherapy-free” protocols. In the ongoing phase III clinical trials NCT04530565, the efficacy of conventional treatment with chemotherapy and corticosteroids along with a tyrosine kinase inhibitor (TKI) is being compared to that of the same regimen augmented with blinatumomab. The primary objective is to compare OS following induction with corticosteroids + TKI + blinatumomab versus induction with corticosteroids + TKI + chemotherapy. The outcomes of this study may help determine whether the combination of corticosteroids, TKI, and blinatumomab is more effective than the standard of care. Moreover, this “chemotherapy-free” approach may reduce the toxicity and side effects associated with chemotherapy while improving patients’ survival rates and quality of life. However, no definitive results have been reported yet, and the study remain in the realm of adult applications. Since the launch of blinatumomab, the timing of drug administration, course of treatment, and scope of application for pediatric patients have all been continuously explores, and methods to reduce drug production costs are also being sought. Research on blinatumomab is continuously being updated, and it is believed that in the future this drug will bring more benefits to pediatric patients.
